# Specific and Novel microRNAs Are Regulated as Response to Fungal Infection in Human Dendritic Cells

**DOI:** 10.3389/fmicb.2017.00270

**Published:** 2017-02-23

**Authors:** Andreas Dix, Kristin Czakai, Ines Leonhardt, Karin Schäferhoff, Michael Bonin, Reinhard Guthke, Hermann Einsele, Oliver Kurzai, Jürgen Löffler, Jörg Linde

**Affiliations:** ^1^Systems Biology/Bioinformatics, Leibniz Institute for Natural Product Research and Infection Biology – Hans Knöll InstituteJena, Germany; ^2^Department of Internal Medicine II, University Hospital of WürzburgWürzburg, Germany; ^3^Septomics Research Centre, Leibniz Institute for Natural Product Research and Infection Biology - Hans Knöll Institute, Friedrich Schiller UniversityJena, Germany; ^4^IMGM Laboratories GmbHMartinsried, Germany; ^5^Institute of Medical Genetics and Applied Genomics, University of TübingenTübingen, Germany; ^6^Center for Sepsis Control and Care, University HospitalJena, Germany; ^7^Institute for Microbiology, University of WuerzburgWuerzburg, Germany

**Keywords:** pathogenic fungi, novel microRNAs, ncRNA, validated target genes, dendritic cells, fungal infection

## Abstract

Within the last two decades, the incidence of invasive fungal infections has been significantly increased. They are characterized by high mortality rates and are often caused by *Candida albicans* and *Aspergillus fumigatus*. The increasing number of infections underlines the necessity for additional anti-fungal therapies, which require extended knowledge of gene regulations during fungal infection. MicroRNAs are regulators of important cellular processes, including the immune response. By analyzing their regulation and impact on target genes, novel therapeutic and diagnostic approaches may be developed. Here, we examine the role of microRNAs in human dendritic cells during fungal infection. Dendritic cells represent the bridge between the innate and the adaptive immune systems. Therefore, analysis of gene regulation of dendritic cells is of particular significance. By applying next-generation sequencing of small RNAs, we quantify microRNA expression in monocyte-derived dendritic cells after 6 and 12 h of infection with *C. albicans* and *A. fumigatus* as well as treatment with lipopolysaccharides (LPS). We identified 26 microRNAs that are differentially regulated after infection by the fungi or LPS. Three and five of them are specific for fungal infections after 6 and 12 h, respectively. We further validated interactions of miR-132-5p and miR-212-5p with immunological relevant target genes, such as *FKBP1B, KLF4*, and *SPN*, on both RNA and protein level. Our results indicate that these microRNAs fine-tune the expression of immune-related target genes during fungal infection. Beyond that, we identified previously undiscovered microRNAs. We validated three novel microRNAs via qRT-PCR. A comparison with known microRNAs revealed possible relations with the miR-378 family and miR-1260a/b for two of them, while the third one features a unique sequence with no resemblance to known microRNAs. In summary, this study analyzes the effect of known microRNAs in dendritic cells during fungal infections and proposes novel microRNAs that could be experimentally verified.

## 1. Introduction

Due to their increasing number, invasive fungal infections are gaining growing importance and the medical community has placed increased emphasis on their impact in public health (Richardson and Lass-Flörl, 2008). Reasons for rising incidences are an increasing number of immunocompromised patients due to transplantations, cancer, and HIV infections (Richardson, 2005; Richardson and Lass-Flörl, 2008). It is estimated that the amount of life-threatening infections by opportunistic fungi exceeds 2,000,000 per year worldwide (Brown et al., 2012). These high numbers are accompanied by a high morbidity of patients and mortality rates of more than 40% in severely immunocompromised patients, such as allogeneic stem cell transplant recipients and patients with acute leukemia, suffering from invasive aspergillosis (Pfaller and Diekema, 2007; Brown et al., 2012; Montagna et al., 2013). Invasive candidiasis, mostly caused by *Candida albicans*, and invasive aspergillosis, mostly caused by *Aspergillus fumigatus*, are the main types of invasive fungal infections (Pagano et al., 2007; Pappas et al., 2010). However, the regulatory response of the immune system to fungal infections has not been completely understood yet.

MicroRNAs are short (≈ 22 nt) non-coding RNAs that post-transcriptionally regulate the gene expression by RNA silencing or RNA degradation. They originate from primary transcripts (pri-microRNAs), which are processed into hairpin structures (precursors, pre-microRNAs) by the Drosha enzyme. After export from the nucleus into the cytoplasm, the pre-microRNAs are then cleaved by the Dicer enzyme into short duplexes of ≈ 22 nt length. One strand of the duplex forms the microRNA-induced silencing complex (miRISC) together with the Argonaute protein and several other proteins (Finnegan and Pasquinelli, 2013). Guided by the microRNA, the miRISC binds to a target mRNA in its 3′-UTR, thereby inducing mRNA destabilization or translational repression (Chekulaeva and Filipowicz, 2009). In a few cases, the binding can also happen in the 5′-UTR or in the coding region (Moretti et al., 2010).

MicroRNAs are involved in many physiological processes like cell differentiation and organ development (Zhao and Srivastava, 2007) but also play crucial roles in the development and functioning of the immune system (Carissimi et al., 2009; Xiao and Rajewsky, 2009). They contribute to the regulation of the immune system by influencing the function and differentiation of various immune cells of both the innate and the adaptive immune responses (Gracias and Katsikis, 2011). The innate immune system, mainly neutrophilic granulocytes and resident macrophages represent the first line of defense against invasive fungal infections. Denditic cells (DCs) are key for the immune defense against fungi as they build a bridge between the innate and the adaptive immune system (Morton et al., 2011). These antigen-presenting cells are involved in pathogen recognition and are solely able to prime naïve T cells in the lymph nodes, thereby determining the fate of the immune response (Wüthrich et al., 2012). Thus, an extended understanding of the regulations may give rise to new therapeutic approaches in anti-fungal therapy.

In this study, we investigate *in vitro* the role of microRNAs in response to fungal infections by *C. albicans* and *A. fumigatus*. Thus, we contribute new knowledge to the role of microRNAs in the response to fungal infections. In a previous study, Monk et al. (2010) analyzed the regulation of microRNAs in murine macrophages when encountering *C. albicans*. In contrast, our study is directly based on human cells instead of model organisms. Moreover, we apply next-generation sequencing of small RNA instead of microRNA microarrays, which allows studying microRNA expression at higher precision and depth and enables the prediction of novel microRNAs (Pritchard et al., 2012). Das Gupta et al. (2014) examined the expression of miR-155 and miR-132 in human DCs and their *in vitro* precursors using *A. fumigatus* and lipopolysaccharides (LPS) as stimuli. In comparison, we are not limited to a previous selection of microRNAs, as we use RNA-sequencing techniques. In this way, we consider all known human microRNAs and thus we may get a broader view on the regulatory mechanisms in DCs.

In conclusion, this study provides insights on the regulatory role of microRNAs in DCs during fungal infections. Based on differential expression and subsequent validation experiments, we demonstrate the fine-tuning effect of selected microRNAs on their immune-relevant targets. Additionally, we discovered and validated novel microRNAs. Therefore, the results of this study contribute to a more comprehensive understanding of the anti-fungal immune response and thus may support the identification of drug targets and the development of novel therapies.

## 2. Materials and methods

### 2.1. *Aspergillus* culture

The fungal strain *A. fumigatus* ATCC 46645 (American Type Culture Collection, LGC Standards) was used for all experiments. Germ tubes were prepared as previously described (Mezger et al., 2008) and inactivated by incubation in 100% Ethanol for 45 min.

### 2.2. *Candida* culture

Wild-type *C. albicans* SC5314 was maintained as previously described (Leonhardt et al., 2015). For experiments, colonies were transferred to M199 medium (PAA), pH 4 and cultured at 30°C to stationary phase. Germ tubes were induced by culturing in M199 medium, pH 8 for 1 h at 37°C. Germ tubes were inactivated by washing them in phosphate-buffered saline (PBS) and followed by incubation in PBS containing 0.1% thimerosal (Sigma-Aldrich) for 1 h at 37°C with shaking. Afterwards, germ tubes were washed five times and then re-suspended in RPMI-1640. Killing was confirmed by plate counts on YPD (2% D-glucose, 1% peptone, 5% yeast extract, Roth) agar plates.

### 2.3. Generation and co-culture of DCs

DCs were generated from PBMCs as previously described (Mezger et al., 2008). Briefly, PBMCs were isolated from completely independent healthy volunteers by ficoll (Bicoll Seperation, Biochrom AG) density gradient centrifugation. Magnetic activated cell sorting with paramagnetic CD14-beads (Miltenyi Biotec) was used to further separate monocytes. Monocyte-derived dendritic cells (DCs) were generated in RPMI-1640 supplemented with 10% FBS (Sigma Aldrich), 120 mg/l Refobacin (Merck), 10 ng/ml IL-4 (Miltenyi Biotec) and 100 ng/ml GM-CSF (Bayer Healthcare) for 6 days.

Co-cultivation experiments of DCs with *A. fumigatus* or *C. albicans* were performed with a multiplicity of infection (MOI) of 1 on day 6 for 6 h and 12 h. LPS (1 μg/ml, Sigma Aldrich) was used in the indicated concentration. Four independent biological replica (no technical replica) were performed. Stimulation of DCs was monitored by flow cytometry. Induction of the DC surface markers CD83 and CD86 was significant for *A. fumigatus, C. albicans* and LPS after 6 and 12 h, respectively. Induction of HLA-DR was significant after 6 h (Data not shown). Furthermore, quantification of secreted protein levels by multiplex ELISA assays demonstrated activation as well.

### 2.4. MicroRNA sequencing

MicroRNA sequencing was performed as previously described (Steinhilber et al., 2015). Briefly, total RNA was isolated using the miRNeasy Mini Kit (Qiagen, Hilden, Germany). RNA samples had a RNA integrity number (RIN) of 8.9 ± 0.9 determined by Agilent Bioanalyzer. 500 ng total RNA, determined by Nanodrop 1000 (Thermo Scientific), was used to generate small RNA sequencing libraries by TruSeq Small RNA Library Prep Kit (Illumina, San Diego, CA) according to the manufacturer's instructions. MicroRNA enriched fractions were isolated from E-Gel (Invitrogen) and quantified by Qbit Instrument (Invitrogen). Equimolare amounts were loaded on a GAIIx single read flow cell (Illumina) and cBot instrument (Illumina) and the TrueSeq Cluster Generation Kit (Illumina) was used to generate clusters. A single 32 nt sequencing run was performed to obtain microRNA sequences. In a next step, barcode sequences were identified by a 7 nt sequencing run. Fastq files were obtained by using CASAVA1.7 (Illumina) and the Fastq ASCII qualities were transferred from Illumina v1.3 to Sanger scale with FastQ Groomer (Cock et al., 2010). Linker sequences were removed by fastx clipper (http://hannonlab.cshl.edu/fastx_toolkit/index.html). The microRNA data has been deposited in the GEO NCBI database (Edgar et al., 2002) (accession code GSE80014).

### 2.5. Short-read mapping and counting

The sequenced reads were mapped to known microRNA precursors, downloaded from miRBase (version 20) using the tool “bowtie” Langmead et al. (2009) (version 0.12.9) with the parameters -v 1 -a -p 1 –best –norc. Afterwards, only those reads were counted which were aligned to the positions of the mature microRNAs in the corresponding precursor sequences, allowing a deviation of 2 nt upstream or downstream. If a read had multiple hits, then the count of this read was divided by the number of hits. In this way, we split the impact of a multiple-hit read and avoid over- or underestimation of microRNAs with very similar sequences. Finally, the counts were rounded to integer numbers. Of the 2,578 mature microRNAs, those exhibiting raw counts fewer than 10 in all samples were discarded to remove noise, leaving 463 microRNAs for analysis. The counts were normalized to “counts per million,” where the counts of each sample are divided by the sum of all counts of each sample (i.e., the library size) and are multiplied by one million.

### 2.6. mRNA analysis

DCs of four independent donors were harvested after 6 and 12 h co-culture with *A. fumigatus, C. albicans* and LPS and analyzed with Affymetrix whole genome expression arrays. RNA samples had a RNA integrity number (RIN) above eight determined by Agilent Bioanalyzer. RNA samples were hybridized to an Affymetrix HG-U219 array plate. Scanned images were subjected to visual inspection to control for hybridization artifacts and proper grid alignment; these were analyzed with AGCC 3.0 (Affymetrix) to generate CEL files. The resulting files, containing a single intensity value for each probe region delineated by a grid on each array image, were imported into Partek Genomics Suite (version 6.6, Partek, St. Louis, MO) for probe set summarization and statistical analysis. Model based Robust Multichip Analysis (Irizarry et al., 2003) was performed for probe set summarization to obtain a single intensity value representing transcript abundance for each probe set and thus enable comparisons between arrays, by normalizing and logarithmically transforming array data and stabilizing variance across the arrays. Microarray data has been deposited in the GEO NCBI database (Edgar et al., 2002) (accession codes GSE69723 and GSE77969).

### 2.7. Differentially expression analysis

Differentially expressed microRNAs (DEMs) were determined using the R package “limma” (Smyth, 2005) (version 3.28.19). Limma uses linear models and moderated t-statistics to detect differential expression. It provides the “voom” function which transforms sequencing data, so that linear modeling can be applied (Law et al., 2014). The *p*-values were adjusted according to the false discovery rate (FDR) (Benjamini and Hochberg, 1995). MicroRNAs exhibiting an adjusted *p*-value < 0.05 were considered as differentially expressed.

To identify differentially expressed mRNAs in the microarray data, also the R package “limma” was used. Those mRNAs with both an FDR-adjusted *p*-value < 0.05 and an absolute fold-change of at least two were considered as significantly differentially expressed.

### 2.8. Target genes of the fungal-specific microRNAs

Experimentally validated microRNA targets were obtained from the database miRTarBase (Hsu et al., 2014) (release 4.5). miRTarBase contains microRNA-target-interactions (MTIs) from manually curated literature search. The MTIs from miRTarBase are categorized into functional and non-functional, where each can be supported by strong or weak experimental evidences. Reporter assays, western blots, and qPCRs are regarded as strong support, while support by microarrays, sequencing data, pSILAC, or other methods is considered as less strong. To get the most reliable information, we filtered the target genes to keep only the functional MTIs with strong support.

MicroRNA targets were further predicted with the tools miRanda (Enright et al., 2003) (version 3.3a) and MirTarget2 (Wang and El Naqa, 2008; Wong and Wang, 2015). MiRanda scans the 3′-UTR of mRNA sequences for possible binding sites based on sequence complementarity and also calculates the free energy of the binding. It was used with the parameters --strict to allow only strict seed pairing, and -en 20, which sets a threshold for the free energy to −20 kcal/mol. Besides the microRNA sequences, miRanda requires the 3′-UTR sequences as input. They were downloaded from Ensembl Biomart (Kinsella et al., 2011) (version 77), covering 18,749 genes.

MirTarget2 uses a support vector machine which was trained with a broader range of features including sequence conservation, the secondary structure of the target site, sequential properties of both the microRNA and the target site, and the location of the binding site within the 3′-UTR. The algorithm MirTarget2 is part of the online resource miRDB (http://www.mirdb.org/), where the microRNA sequences were submitted to receive predicted target genes.

### 2.9. Validation of predicted microRNA targets

All RNA interference experiments were performed as previously described (Mezger et al., 2008). Briefly, DCs were electroporated (EPI 2500, Dr. L. Fischer) with either short interfering single-stranded RNA inhibiting miR-132-5p, miR-212-5p or non-silencing, random RNA (ThermoFisher) at 340 V for 10 ms on day 5 after isolation and then incubated at 37°C and 5% CO_2_ for 24 h in culture medium. MicroRNA quantification was performed after stimulation with either *A. fumgiatus, C. albicans* or LPS, as previously described (Das Gupta et al., 2014). RNA was isolated by using miRVana microRNA isolation kit (Thermo Fisher Scientific) according to manufacturer's instructions. Specific cDNA was generated from 100 ng template. Subsequent quantification of these specific microRNAs was performed by quantitative real-time PCR (qPCR) with TaqMan probes as described by the manufacturer (MicroRNA RT Kit and microRNA-specific TaqMan MicroRNA Assays, both Thermo Fisher Scientific). cDNA synthesis of mRNA was performed with First Strand cDNA synthesis Kit (Thermo Fisher Scientific). Quantitative real-time PCR was performed on a StepOne Plus (Applied Biosystem) with iTaq Universal SBYR Green Supermix (BioRad) and the primers for *BTN3A2* (NM_001197248.2) fw: ACAGAGCGGGAAATAAGCCT, rv: AACAAGGTGGAGCCTCATCTG, *FKBP1B* (NM_004116.3) (AGATCGAGACCATCTCCCCC), rv: AAGCTCATCTGGGCTGCAC, *KLF4* (NM_004235.4) fw: TACCAAGAGCTCATGCCACC, rv: CGCGTAATCACAAGTGTGGG, *SPN* (NM_001030288.2): fw CTTATCAGCCGAGCCGGTCC, rv: GTGTCTGCACTGCTGTTGTG, *TRIM22* (NM_001199573.1) fw: AGATCTCCAGCGGAGGTTGA, rv: AACTTGCAGCATCCCACTCA, *IFITM2* (NM_006435.2) fw: ATCCCGGTAACCCGATCAC, rv: CTTCCTGTCCCTAGACTTCAC, *ALAS1* (NM_000688.5) fw: GGCAGCACAGATGAATCAGA, rv: CCTCCATCGGTTTTCACACT (Sigma Aldrich) were used in a concentration of 0.5 μM at a total reaction volume of 20 μl.

### 2.10. Western blotting

Cells were re-suspended in lysis buffer (6.65 M Urea (Sigma), 10% Glycerin, 1% SDS, 10 mM Tris (Carl Roth GmbH), pH 6.8), sonicated for 15 s (UP50H, Hielscher) and proteins quantified using the DC Protein Assay (BioRad). Cell lysates were subjected to sodium dodecyl sulfate polyacrylamide gel electrophoresis (SDS-PAGE) and transferred to nitrocellulose membranes. Membranes were incubated with rabbit anti-KLF4, anti-IFITM2 (Cell Signaling), goat anti-SPN and anti-FKBP1B (R&D systems) and mouse anti-β-Actin (Sigma Aldrich) antibodies over night at 4°C or for 2 h at room temperature, respectively. Horseradish-conjugated anti-rabbit, anti-mouse (Cell Signaling) and anti-goat (R&D systems) IgG antibodies were used as secondary antibodies. Signals were visualized by ECL reaction with Clarity^TM^ ECL Western Substrate (BioRad) or Pierce^TM^ ECL Western Blotting substrate (Thermo Scientific) and analyzed using ImageJ software (ImageJ version 1.47, National Institutes of Health).

### 2.11. Discovery of novel microRNAs

Previously unknown microRNAs were discovered by using the tool miRDeep2 (version 2.0.0.5) (Friedländer et al., 2012). The mapper module of miRDeep2 was run with the parameters -d -e -h -i -j -m -u -v to perform sequence preprocessing and to map the reads to the human genome (hg19). For the miRDeep2 module, the input data were the precursor and mature microRNA sequences of *Homo sapiens* from miRBase, the mapping of the mapper module, and the mature microRNA sequences of the hominoids *Gorilla gorilla, Pan paniscus, Pongo pygmaeus, Pan troglodytes*, and *Symphalangus syndactylus* as input from related species. The latter are used by miRDeep2 to search for seed sequence conservation, which is included into the scoring. According to the recommendation of Friedländer et al. (2012), we considered only the predictions with a signal-to-noise ratio of 10 or higher for further analysis. We further filtered the predicted microRNAs using a randfold *p*-value of 0.05 as cutoff, which suggests stable secondary structures, and kept only predictions without a “Rfam alert,” which indicates that a rRNA or tRNA was found instead of a microRNA. Subsequently, we defined predictions as high-confidence, if the 3p as well as the 5p mature microRNA were found at least in two time points or two different infections (*A. fumigatus, C. albicans*, or LPS). Overlapping high-confidence predictions were resolved by keeping those with the highest scores. Finally, the counts of the predicted mature microRNAs were determined for each sample.

To identify sequence similarities between novel and known microRNAs, we used the tool BLAST (version 2.2.31) with the known human mature microRNAs from miRBase (version 20) and the validated novel microRNAs as input. For multiple sequence alignment, ClustalW (Larkin et al., 2007) (version 2.1) was used with default parameters. The phylogenetic tree was plotted with TreeDyn (Chevenet et al., 2006) (version 198.3) on the Phylogeny.fr server (Dereeper et al., 2008) using default settings.

### 2.12. Validation of predicted novel microRNAs

RNA extraction was performed using the miRNeasy Kit (Qiagen). 500 ng of RNA was applied to perform cDNA synthesis using the miScript II RT Kit (Qiagen). qRT-PCR was performed on a LightCycler480 System (Roche, Mannheim, Germany) using the miScript SYBR Green PCR Kit (Qiagen) according to the manufacturer's instructions. Standard curves for each amplified transcript were generated to obtain the PCR efficiency. CP-values were determined by the LightCycler Software 480 (Roche). Expression levels of each sample were detected in triplicate reactions. RNU6B (content of the Hs_RNU6B_2 miScript Primer Assay, Qiagen) was used as reference gene. Relative expression levels of each target microRNA were calculated applying the efficiency-corrected equation by Pfaffl et al. (2002) using qbase PLUS v2.4 (Biogazelle). For the detection of predicted microRNAs oligonucleotides with the respective sequence of the mature microRNA were used as forward primer. The universal reverse primer was included in the miScript SYBR Green PCR Kit. The sequences of the oligonucleotides are listed in the Supplementary Table 1. Validation experiments were performed on specimens from independent donors.

### 2.13. Ethics

This study, using whole blood specimens obtained from human healthy volunteer donors, was approved by the Ethics Committee of the University of Würzburg (approval 302/12). Data analysis was conducted anonymously.

## 3. Results

### 3.1. MicroRNA signatures are specific for fungal infections

In order to study the expression of human microRNAs during fungal infection, we analyzed next-generation sequencing data of human dendritic cells (DCs) infected with *A. fumigatus* and *C. albicans* germ tubes. To compare the expression of microRNAs during fungal infection with bacterial infection, we simulated bacterial infection by adding lipopolysaccharides (LPS) to DCs. As a control for all infected DCs, we used mock infected samples.

**Figure 1:**
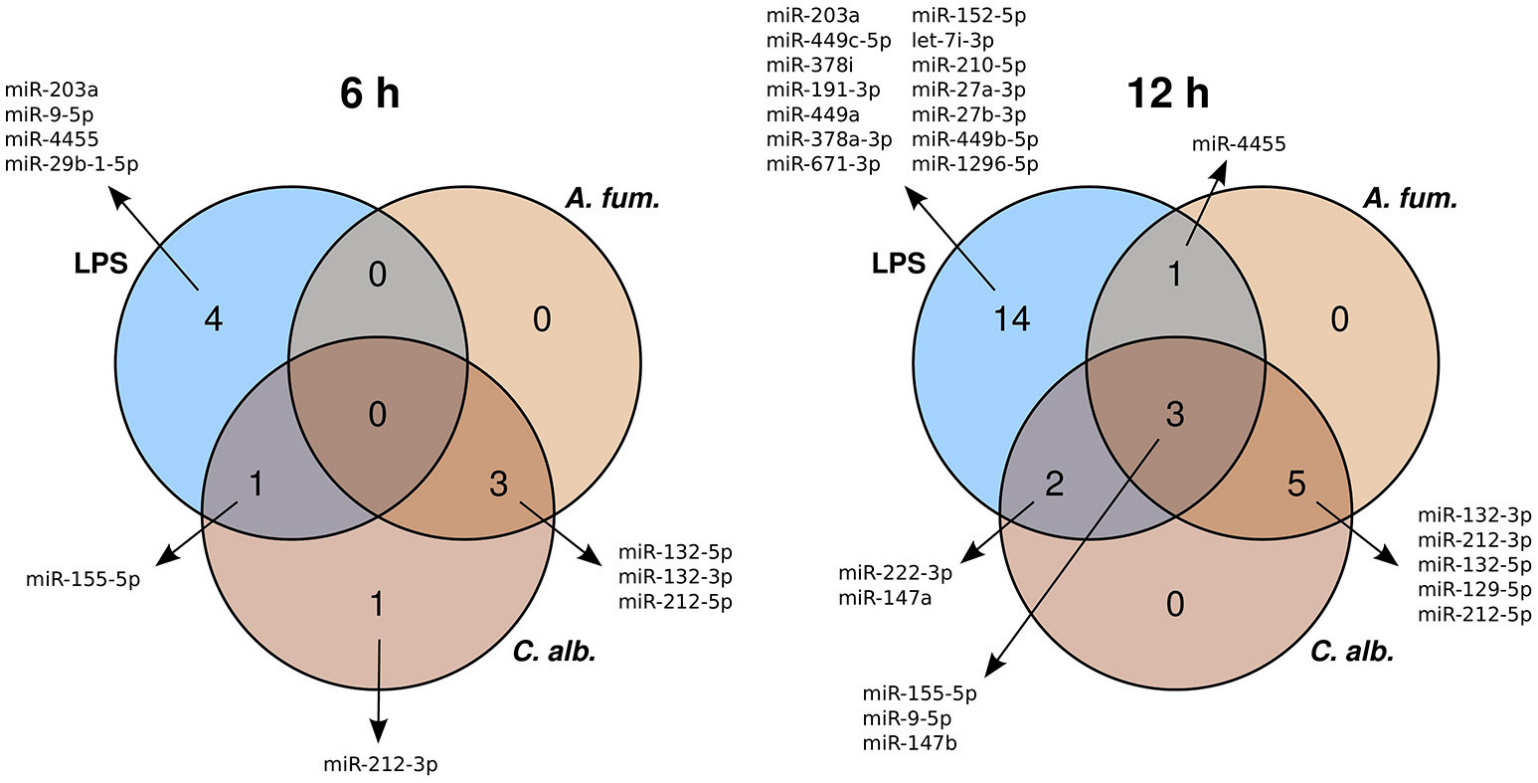
**Venn diagrams of the differentially expressed microRNAs of the treatments with *A*. *fumigatus*, *C. albicans*, or LPS**.

Six hours post infection, we identified three, five, and five differentially expressed microRNAs (DEMs) for *A. fumigatus, C. albicans*, and LPS, respectively (Supplementary Table 2). Twelve hours post infection 9, 10, and 20 DEMs were detected. The vast majority of the DEMs were up-regulated, while four microRNAs showed a down-regulation. Several DEMs are overlapping between the conditions (*A. fumigatus, C. albicans* germlings, and LPS) and the time points (6 and 12 h) (Figure 1).

**Figure 2:**
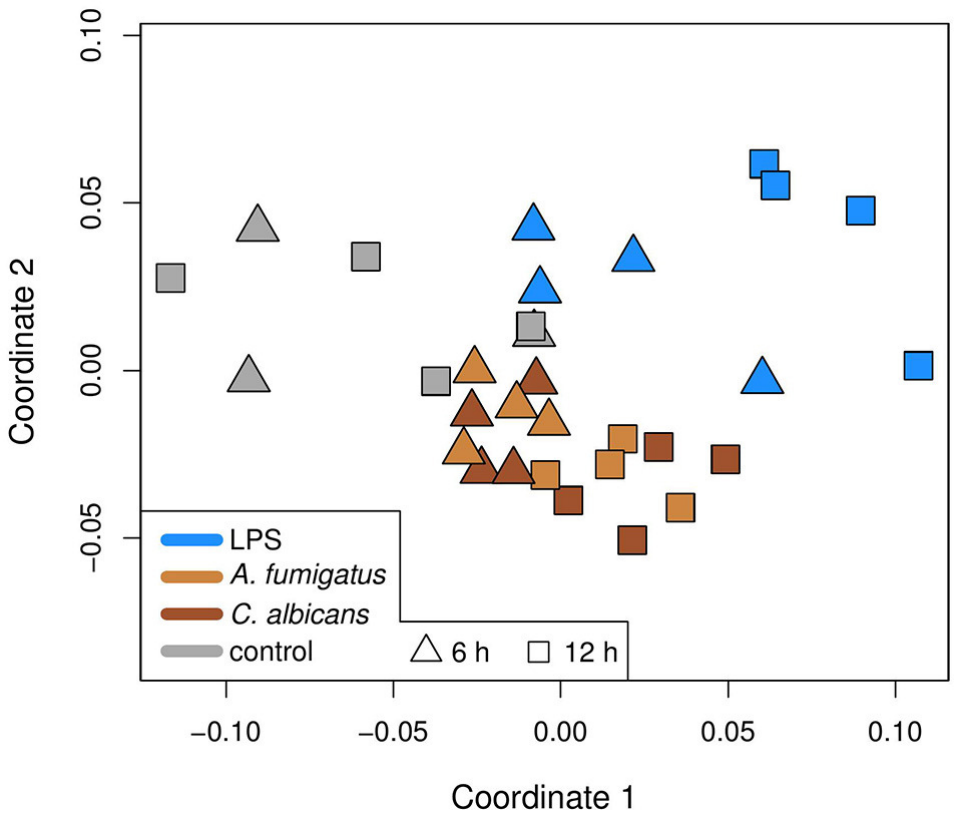
**Multidimensional scaling (MDS) plot of the samples in this study**. The MDS is based on Spearman correlations, which means that similar samples are close to each other, while different samples are distant. Colors represent the treatment of a sample and the shapes the time point. Infection is different to control and bacterial infection is different to fungal.

We clustered the expression data of all 26 unique DEMs by using multidimensional scaling (MDS), which is a dimension reduction method and thus allows visualizing of similarities between samples. The clustering is based on Spearman correlations, which means that samples with high correlation coefficients are close to each other in the plot. The MDS plot (Figure 2) shows the grouping of the data. There is a separation between fungal and LPS samples, while the samples of the fungal species are mixed. Moreover, the 6 h samples of all three conditions are closer to the control samples, indicating higher similarities than the 12 h samples.

To select DEMs that are specific for the response to fungal infections, we determined which DEMs were differentially regulated for *A. fumigatus* and *C. albicans* together, but not for LPS. In this way, we found three DEMs (miR-132-3p, miR-132-5p, miR-212-5p) for the 6 h and five DEMs (miR-129-5p, miR-132-3p, miR-132-5p, miR-212-3p, miR-212-5p) for the 12 h samples (Figure 1). While miR-132-3p has been linked to *A. fumigatus* infections before (Das Gupta et al., 2014), it is also involved in different immune processes together with miR-212-3p (Wanet et al., 2012). In contrast, this is the first study which identified miR-132-5p and miR-212-5p to play major roles in the immune regulation. Besides that, the microRNA miR-129-5p has been associated to different cancer types (Li et al., 2013; Yu et al., 2013).

### 3.2. Fungal-specific microRNAs target immune related genes

As microRNAs play an important role in post-transcriptional gene regulation, we analyzed their target genes. There are many tools for predicting the microRNA targets, where each one considers different features of the microRNA-target-interaction (MTI). Therefore, we decided to use multiple sources to cover a broader range of potential target genes. We used the database miRTarBase to obtain a list of experimentally validated MTIs. Additionally, we used miRanda and MirTarget2 to predict microRNA targets. With these three sources, we determined target genes for the fungal-specific DEMs. While the number of their validated MTIs is below twelve, the prediction of target genes yielded several hundred candidates (Table 1).

**Table 1:** **The number of target genes obtained from different resources**.

**microRNA**	**miRTarBase**	**miRanda**	**MirRarget2**
miR-132-3p	11	302	379
miR-132-5p	0	543	41
miR-129-5p	3	168	896
miR-212-3p	8	383	382
miR-212-5p	0	1857	325

*While miRanda and MirTarget2 predict putative microRNA targets, miRTarBase provides experimentally validated target genes*.

Since microRNA target prediction is a difficult task, which often leads to a high number of false positives Hamzeiy et al. (2014), it is important to filter for the most promising and interesting candidates. As shown by Eichhorn et al. (2014), mRNA destabilization is the main mode of repression by microRNAs. Therefore, we can assume that during time course of infection the expression of potential target genes is inversely correlated to the expression of their microRNA regulators. In accordance with this assumption, we used microarray data of the same conditions and time points to determine which of the target genes are significantly down-regulated, since all of the fungal DEMs are up-regulated in both time points. In a first filtering step, we kept only those target genes that show down-regulation for *A. fumigatus* or *C. albicans*. The second filtering step comprises a correlation analysis. We calculated the Pearson correlation of the microRNA expression values and the expression intensities of the filtered target genes across all samples. Genes with a correlation coefficient of -0.5 or less were retained as high-confidence targets. This correlation threshold has been used successfully in previous microRNA studies (Fu et al., 2012; Summerer et al., 2015). Applying both filters left 19 unique target genes. Some of them are regulated by multiple microRNAs (Tables 2, 3).

**Table 2:** **Correlation coefficients between the expression of fungal DEMs for 6 h and their high-confidence targets**.

**6 h**					
**miR-132-3p**		**miR-132-5p**		**miR-212-5p**	
SAP30L	−0.750	FKBP1B	−0.866	KLF4	−0.832
ATP10D	−0.881			DHTKD1	−0.801
ABCG1	−0.813			DNPEP	−0.790
BARD1	−0.556			TRIM22	−0.531
TTC39C	−0.859				

**Table 3:** **Correlation coefficients between the expression of fungal DEMs for 12 h and their high-confidence targets**.

**12 h**									
**miR-132-3p**		**miR-132-5p**		**miR-129-5p**		**miR-212-3p**		**miR-212-5p**	
MTMR1	−0.788	BTN3A2	−0.663	FAM46A	−0.545	SP110	−0.778	KLF4	−0.748
SP110	−0.799	FKBP1B	−0.934	ADD3	−0.588	MTMR1	−0.863	DHTKD1	−0.865
TTC39C	−0.773			CASP6	−0.606			DNPEP	−0.828
				CCDC170	−0.516			SPN	−0.772
								TRIM22	−0.690
								IFITM2	−0.795

The resulting set of target genes includes genes playing a role in immune response. For example, for miR-132-5p, these genes are *BTN3A2*, which is associated to the stimulation of the adaptive immune response in DCs Simone et al. (2010), and *FKBP1B* that is associated to T cell proliferation in mice (Dubois et al., 2003) and is connected to the response to viral infections (Krishnan et al., 2008; Carbajo-Lozoya et al., 2012).

### 3.3. MicroRNAs fine-tune the gene expression in dendritic cells

In order to confirm and analyze the predicted and filtered microRNA targets, we selected multiple predicted MTIs for validation via silencing of microRNA and qPCR analysis of their potential target genes. We decided to concentrate on the microRNAs miR-132-5p and miR-212-5p for two reasons: First, only little is known about the roles of these two mircoRNAs in the anti-fungal response. Second, both microRNAs have the most immune-related target genes in their filtered lists (see above). The immunological relevant targets of miR-212-5p are *KLF4*, which has been shown to be involved in the response of DCs to fungal infections (Czakai et al., 2016) and to be important for inflammatory immune response (Alder et al., 2008; Rosenzweig et al., 2013), and *SPN*, a surface molecule of various immune related cells, e.g., T lymphocytes, thymocytes, monocytes, and neutrophiles (Remold-O'Donnell et al., 1987). Moreover, both *TRIM22* and *IFITM2* play a role in the anti-viral immune response (Brass et al., 2009; Singh et al., 2011).

**Figure 3:**
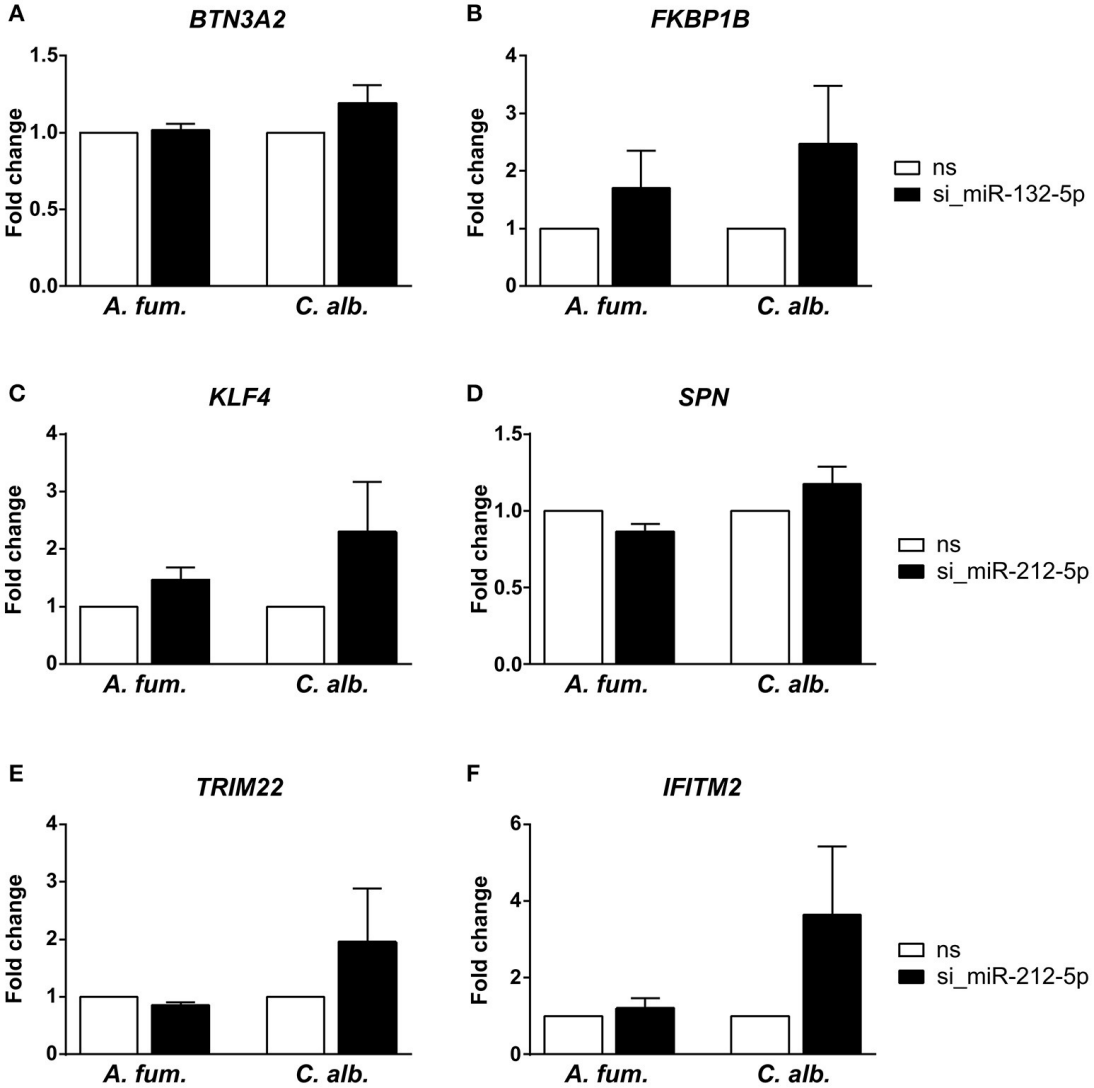
**Experimental analysis of predicted microRNA-target-interactions by silencing of the microRNAs (A,B)** miR-132-5p and **(C–F)** miR-212-5p using siRNA. Target gene expression was measured with quantitative real-time PCR. We observed a trend toward up-regulation for the target genes, although this regulation is not statistically significant. The infection with *C. albicans* led to a stronger up-regulation than the infection with *A. fumigatus* (ns = random non-silencing control siRNA sample).

**Figure 4:**
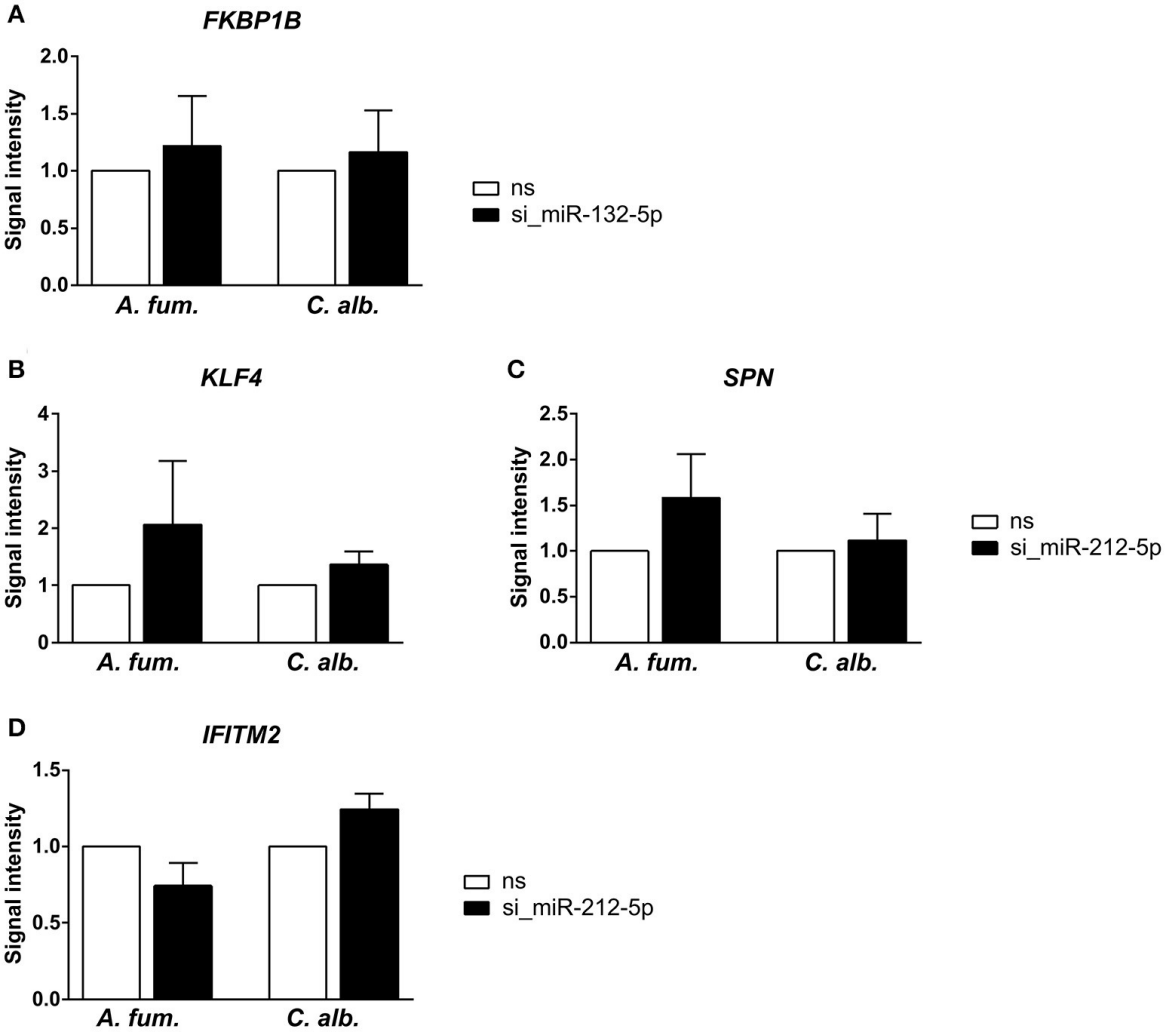
**Further indication for microRNA-target-interactions were collected via western blots and silencing of the microRNAs (A)** miR-132-5p and **(B–D)** miR-212-5p. Similarly to the PCR experiments, the up-regulation of the predicted target genes is not statistically significant, but a trend toward up-regulation can be found. The up-regulation is stronger for the *A. fumigatus* infection compared to the *C. albicans* infection.

We silenced miR-132-5p and miR-212-5p in separate experiments in DCs generated under identical conditions and in accordance to the co-incubation settings using microRNA inhibitors. The validation results (Figure 3) revealed that the regulatory effects of the microRNAs are weaker than suggested by the microarray data. However, an up-regulation of the target genes (*BTN3A2, FKBP1B, KLF4, SPN, TRIM22*, and *IFITM2*) is visible when the corresponding microRNAs are silenced. Infections with *C. albicans* led to an increased up-regulation compared to *A. fumigatus* infections for all target genes. Additionally, for the genes *BTN3A2, SPN*, and *TRIM22*, no up-regulation is visible for *A. fumigatus* infections with silenced microRNAs in the qPCR experiments. Although the expression differences between the silenced and the non-silenced measurements are not statistically significant, a trend toward increased expression intensities in case of microRNA silencing can be found. We further analyzed the expression changes of *FKBP1B, KLF4, SPN*, and *IFITM2* on protein level by performing western blot experiments. Similar to the qPCR results, a trend toward up-regulation on microRNA silencing can be found (Figure 4). In contrast to the qPCR data, the increase of protein amount is higher for *A. fumigatus* infections than for *C. albicans* infections, with the exception of *IFITM2*. The validation experiments of both qPCR and western blot indicate that the regulation by the microRNAs happens on a level of fine-tuning.

### 3.4. Discovery of novel microRNAs

Next-generation sequencing not only allows to study gene expression with high accuracy, but also the identification of so far unknown genes (Linde et al., 2015) as well as microRNAs (Dannemann et al., 2012). To our knowledge, this is the first study which performs next-generation sequencing of human microRNAs during fungal infection. Therefore, we scanned the RNA-Seq data for previously unknown microRNAs. We applied the tool miRDeep2 (Friedländer et al., 2012) to the control and the LPS samples, as well as to the fungal data comprising the pooled data of both fungal species. The miRDeep2 algorithm works in multiple stages. First, the reads of the microRNA dataset are preprocessed (i.e., clipping of adapter sequence and collapsing identical reads) and mapped to the genome. Next, the algorithm excises potential microRNA precursors from the genome, which is guided by the read mappings. Then, for each precursor sequence, the secondary structure and the energetic stability are predicted. Finally, the precursors are scored.

**Figure 5:**
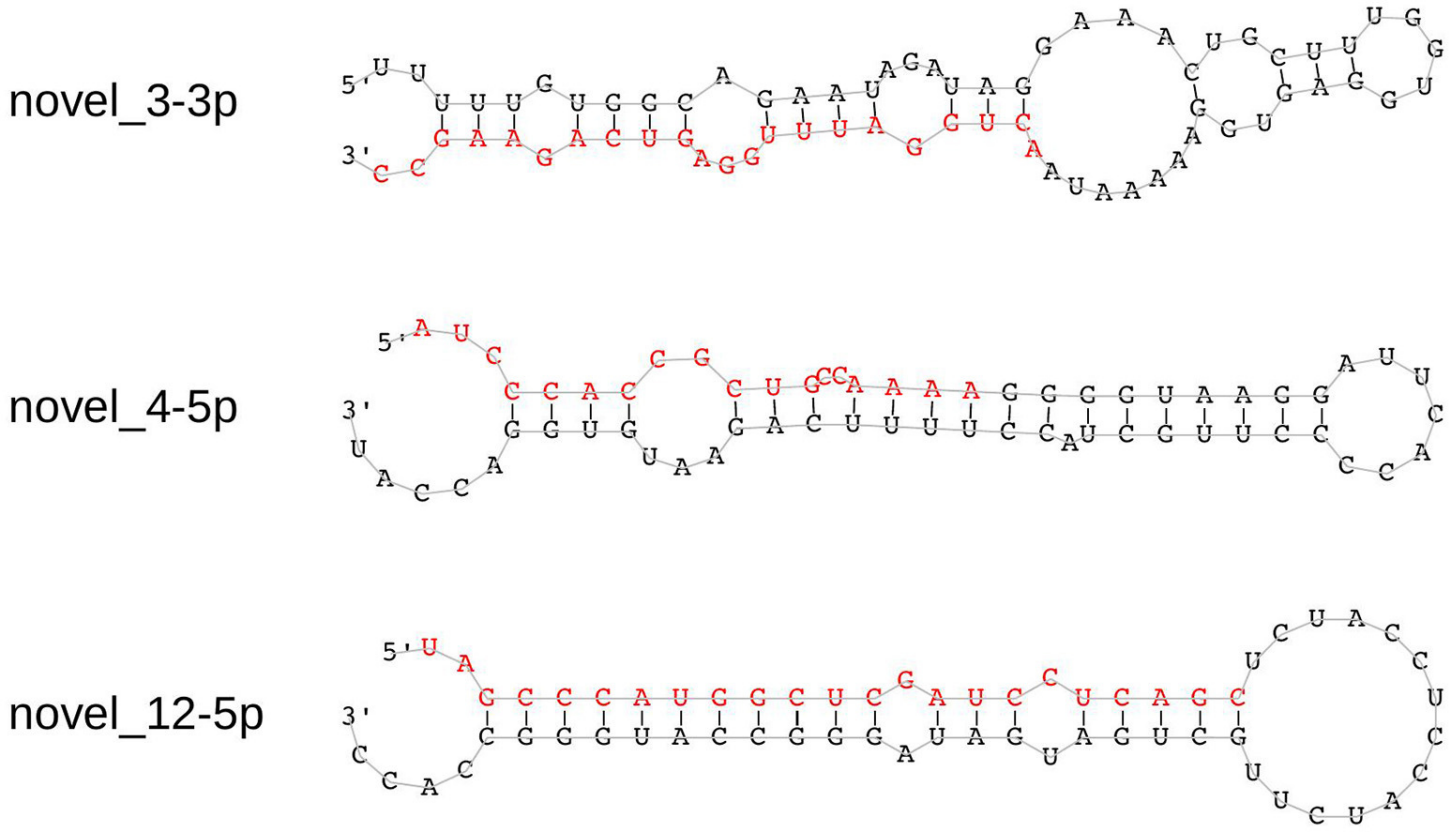
**Hairpin structures of the three experimentally validated novel microRNAs**. The sequences of the mature microRNAs that were predicted by miRDeep2 are shown in red.

**Figure 6:**
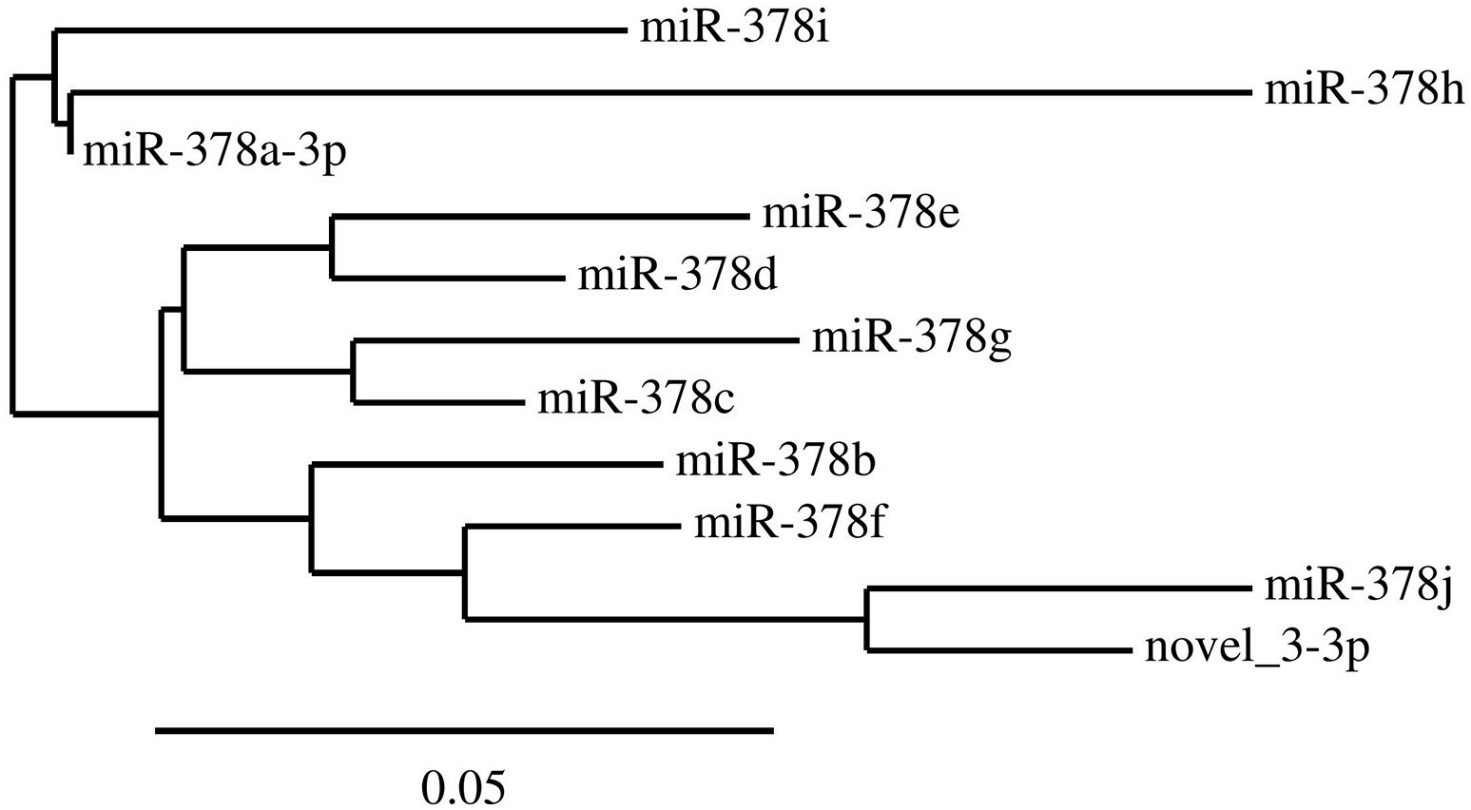
**The phylogenetic tree of the members of the miR-378 family**. The tree was generated by ClustalW and shows the relations between the microRNAs based on their sequences. It indicates that the newly discovered microRNA novel_3-3p is closest related to miR-378j.

**Figure 7:**
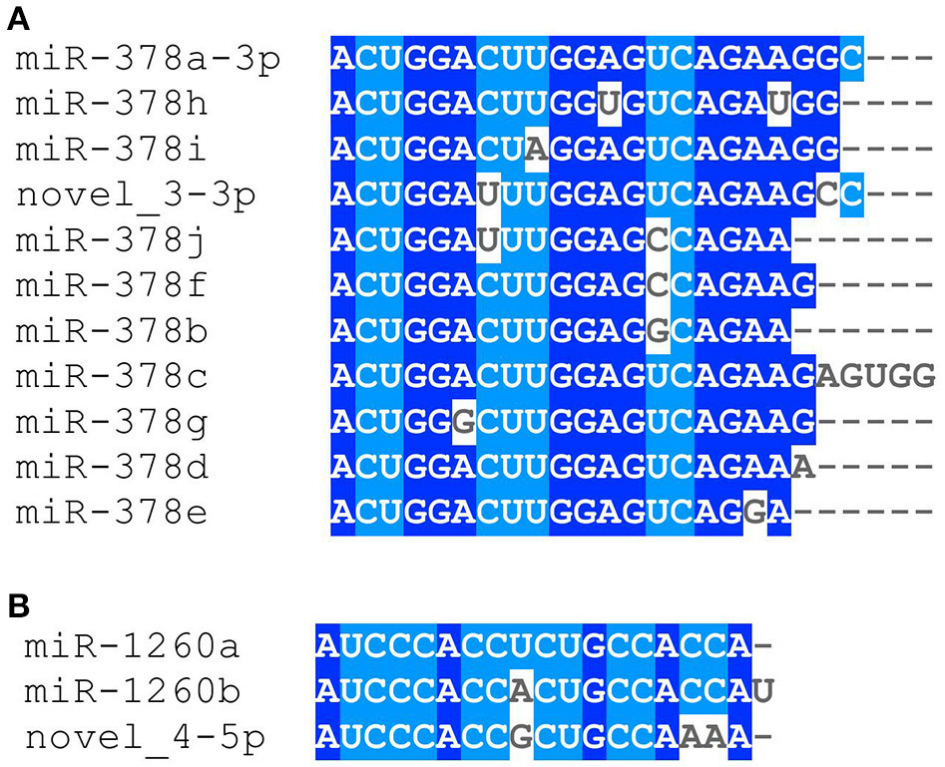
**Alignment of the novel microRNAs (A)** novel_3-3p with the members of the miR-378 family and **(B)** novel_4-5p with miR-1260a/b. The alignment was created with ClustalW.

We identified 21 non-overlapping high-confidence predictions. For each of these predictions, we determined whether the 3p and/or the 5p mature microRNA has counts of at least ten in three or more samples. While seven microRNAs fulfilled this criterion, the remaining ones were discarded. In the course of this study, we name these 7 microRNAs novel_1-5p, novel_3-3p, novel_4-5p, novel_12-5p, novel_18-5p, novel_20-3p, and novel_21-3p. Five of them (novel_3-3p, novel_12-5p, novel_18-5p, novel_20-3p, and novel_21-3p) are located in introns of protein coding genes, where novel_3-3p and novel_21-3p are on the opposite strand of the respective genes. The pri-microRNA of novel_20-3p, however, also overlaps with an exon of gene *WDFY4*. Novel_4-5p is located in an intergenic area and novel_1-5p lies within the 3′-UTR of gene *DDX58* (Table 4 and Supplementary Table 3). Of the seven predicted novel microRNAs, three could be validated by qPCR experiments in independent samples. These are novel_3-3p, novel_4-5p, and novel_12-5p (Figure 5 and Supplementary Table 4). We then used BLAST to compare the sequences of these three microRNAs to the known sequences from miRBase (Supplementary Table 5). The BLAST results revealed a high similarity of novel_3-3p to the sequences of the miR-378 family. Therefore, we performed a multiple sequence alignment using ClustalW to determine which of the miR-378 microRNAs is most closely related to novel_3-3p. The resulting phylogenetic tree visualizes the relations between the sequences (Figure 6). The microRNA miR-378j has the smallest distance to novel_3-3p, indicating highest similarity. Another hint for a close relation to miR-378j is the fact that this microRNA is the only member of the miR-378 family, which has no mismatch in the seed sequence compared to novel_3-3p (Figure 7). The microRNAs of the miR-378 family play a role in rhabdomyosarcoma, myogenesis, and apoptosis (Knezevic et al., 2012; Megiorni et al., 2014).

**Table 4:** **The genomic locations of the mature, precursor, and primary sequences of the validated novel microRNAs**.

**Name**	**Chromosome**	**Strand**	**Mature**		**Precursor**		**Primary**	
			**Start**	**End**	**Start**	**End**	**Start**	**End**
novel_3-3p	chr12	+	29715552	29715573	29715503	29715573	29715482	29715591
novel_4-5p	chr6	−	10662281	10662298	10662236	10662298	10662211	10662318
novel_12-5p	chr19	+	52303197	52303219	52303197	52303258	52303177	52303288

The BLAST results further indicate a connection between miR-1260a/b and novel_4-5p. They share the same seed sequence and, except for one mismatch, the first 15 nt are identical at lengths of 18-19 nt (Figure 7). While miR-1260a was shown to be up-regulated in patients with pelvic organ prolapse (Jeon et al., 2015), miR-1260b plays a role in kidney cancer (Hirata et al., 2013). The seed sequence of novel_12-5p seems to be unique among the known microRNAs and also the BLAST results did not yield any major sequence similarities.

## 4. Discussion

In this study, we present the analysis of the regulatory response of microRNAs of human DCs in reaction to fungal infection and LPS treatment. As a first step, we identified the DEMs for each condition. When visualizing the expression patterns of the DEMs by an MDS (Figure 2) plot, we made two major observations. First, we found that the samples of the fungal species are mixed and are clearly separated from the LPS samples. This finding indicates a similar microRNA regulation in the anti-fungal response. Moreover, this response seems to be specific and different from the reaction to LPS. This indicates that microRNAs might be used as biomarkers discriminating fungal from bacterial infections which is often crucial for treatment decisions in clinical settings. Second, we discovered that for all conditions the 6 h samples were more closely related to the controls than the 12 h samples. This suggests that the regulation in consequence of the treatment was stronger after 12 h than after 6 h. One possible reason for this observation is that microRNA expression has to be induced by a trigger event, which in this study is the treatment with a fungus or LPS. This induction may not be completed after 6 h and thus more microRNAs are up-regulated at 12 h. Both of the above mentioned findings can be confirmed by the Venn diagrams (Figure 1), showing overlaps of the DEMs for the three conditions. In these diagrams, we found that the overall numbers of DEMs are smaller for 6 h compared to 12 h. Second, two peaks in the DEM numbers can be found for both time points: for LPS only as well as for *A. fumigatus* and *C. albicans* together.

The analysis of differential expression revealed similar regulatory patterns for the 3p and the 5p mature microRNA of both mir-132 and mir-212 after 12 h of stimulation. These two microRNAs are relatively closely located in the genome and member of the miR-132/212 cluster. In addition, miR-132-3p and miR-212-3p share the same seed sequence and thus may have many common target genes. In our study, we obtained a similar list of target genes for these two microRNAs (Tables 2, 3). The regulatory patterns of the miR-132/212 cluster suggest a strong involvement of these microRNAs in the anti-fungal response. However, the data also indicated that they are of no major importance for the response to LPS. It has been reported that miR-132-3p and miR-212-3p are involved into the neuron morphogenesis, are down-regulated in several brain-related disorders, and also play a role in regulating immune processes (Wanet et al., 2012). Moreover, Das Gupta et al. (2014) showed an up-regulation of miR-132-3p in DCs in response to *A. fumigatus* stimulation, whereas a stimulation with LPS had no effect on miR-132-3p. These observations are in accordance with our results, which supports the assumption of a regulatory function specific to fungal infections.

As shown in many studies, microRNAs may also serve as biomarkers (Keller et al., 2011; Rupani et al., 2013; Keller et al., 2014; Dix et al., 2015). Their presence/absence or their expression levels can indicate particular conditions of the organism. These associations can be used to improve the diagnosis of a disease or to better predict the prognosis of it. As indicated by out data as well as by the results from Das Gupta et al. (2014), miR-132-3p may be considered as a novel biomarker candidate. Based on data from our study, microRNA profiling might be applied to the diagnosis of other pathogenic fungi or infectious diseases. However, follow-up experiments are required to assess the quality of the presented microRNAs as biomarkers in experiments with a larger sample size and a broader range of fungal infection studies.

We used different tools to determine the target genes of the fungal-specific microRNAs and filtered these lists based on inverse regulation and correlation.The number of predicted target genes is much higher than the number of validated target genes. A reason is that bioinformatic target prediction tools yield high amounts of false positive results, since microRNAs are very short. Additionally, the number of validated targets is closely connected to the factor of how well studied a microRNA is. Validation experiments are usually performed for microRNAs that are relevant for a study, which therefore leads to a bias in the number of verified MTIs. In the presented results, the most immunological relevant target genes were identified for the microRNAs miR-132-5p and miR-212-5p. These microRNAs are not yet known to be of immunological significance. We performed experiments to validate the predicted interactions with these immune-relevant genes. Although no statistical significance could be found for the expression changes of the selected target genes after microRNA silencing, a trend of up-regulation among these genes was observed. However, the regulatory patterns of the PCR experiments and the western blots are contrary for *A. fumigatus* and *C. albicans*. This finding indicates a considerable influence of post-transcriptional and/or translational regulation as well as of the different stabilities of RNA and proteins. Indeed, it has been reported that the squared Pearson correlation of mRNA and protein levels is only at ≈0.4 (Vogel and Marcotte, 2012). The small extend of up-regulation suggests that the microRNAs perform a fine-tuning of the gene expression and other gene regulatory mechanisms, for example transcription factors, seem to play a larger role for the anti-fungal immune response in DCs. Nevertheless, fine regulatory mechanisms are important for controlling immune processes (Gantier et al., 2007). For example, studies have shown that microRNAs fine-tune the TLR4 signaling and the sensitivity of T cell during their differentiation (Sonkoly et al., 2008; O'Neill et al., 2011).

Although our validation experiments are limited in their extent, we could identify and verify miR-132-5p and miR-212-5p as regulators in DCs during fungal infections. To demonstrate the relevance of miR132-5p and miR212-5p in additional immune cell populations, experiments stimulating human primary natural killer (NK) cells with *A. fumigatus*, followed by subsequent microRNA extraction and qPCR-based quantification of miR132-5p and miR212-5p expression were performed. While expression of miR132-5p did significantly change in NK cells stimulated with *A. fumigatus*, expression of miR212-5p was significantly increased after 6 h of co-cultivation (*p* = 0.036). This observation indicates the general relevance of miR212-5p in various cell populations of the innate immunity. In addition, our previous work shows that in human primary monocytes miR-132 (and also miR-155) was differentially expressed after stimulation with *A. fumigatus*. Interestingly, miR-132 was induced by the fungus but not by LPS in this cell type (Das Gupta et al., 2014). Furthermore, Wanet et al. (2012) reported that miR-212 and miR-132 are expressed in various immune cells, like macrophages, mast cells and lymphatic endothelial cells. However, it remains an open task for future studies to shed light on the role of these microRNAs in other tissues and under different conditions. Also, an analysis of a broader range of microRNA regulations would be of great value.

Beside the analysis of the known microRNAs, we predicted previously undiscovered microRNAs. Three novel microRNAs were experimentally confirmed. The sequence similarities of novel_3-3p to the members of the miR-378 family strongly suggest that we found a new microRNA of this family. In addition, we discovered a striking resemblance of novel_4-5p to the microRNAs miR-1260a/b. For this reason, we strongly believe that the newly identified novel_4-5p is closely related to these two microRNAs. We hope that our discoveries may help the scientific community to reveal new regulatory relationships in the field of microRNA research.

In conclusion, this study provides new insights into the regulatory role of microRNAs in DCs in response to fungal infections by *C. albicans* and *A. fumigatus*. We identified expression patterns that are common for both fungal pathogens and are different from LPS treatment. We performed validation experiments of microRNAs and their predicted target genes revealing fine-tuning regulations by miR-132-5p and miR-212-5p in anti-fungal immune response. Moreover, we successfully discovered and experimentally verified novel microRNAs. While two of them are probably strongly related to known microRNAs, the third one represents a completely new microRNA species.

## Author contributions

AD did the bioinformatic analysis and co-wrote the manuscript. KC and IL conducted the initial experiments, generated the data, and co-wrote the manuscript. KC also performed the validation experiments of the microRNA-target-interactions. KS did the validation experiments of the novel microRNAs and co-wrote the manuscript. MB, RG, HE, OK, JLö, and JLi designed the study, discussed the results, and co-wrote the manuscript.

### Conflict of interest statement

The authors declare that the research was conducted in the absence of any commercial or financial relationships that could be construed as a potential conflict of interest.
